# Predicting the quality of digital mammography from the perspective of detectability of microcalcifications: A Radiomics approach

**DOI:** 10.1002/mp.18061

**Published:** 2025-08-15

**Authors:** Lucas E. Soares, Lucas R. Borges, Renato F. Caron, Denise Y. Nersissian, Marcelo A. C. Vieira

**Affiliations:** ^1^ Department of Electrical and Computer Engineering, São Carlos School of Engineering University of São Paulo São Carlos Brazil; ^2^ Real‐Time Tomography LLC Villanova USA; ^3^ Barretos Cancer Hospital Pio XII Foundation Barretos Brazil; ^4^ Department of Nuclear Physics, Institute of Physics University of São Paulo São Paulo Brazil

**Keywords:** digital mammography, image quality assessment, machine learning, model observer, radiomics

## Abstract

**Background:**

Image quality in mammography is influenced by several technical factors, including radiation dose, exposure parameters, and detector type. Although observer studies are essential for assessing image quality and lesion detectability, they are complex, time‐consuming, and costly, making routine implementation challenging. Traditional objective metrics for quality assessment have limited applicability in medical imaging and often rely on a ground‐truth reference, which is typically unavailable in clinical scenarios. Therefore, methods that assess image quality directly from clinical images, particularly when linked to diagnostic performance, are highly desirable.

**Purpose:**

To propose a method based on radiomic features and machine learning to predict the quality of clinical mammography images in terms of microcalcification detectability, without relying on routine observer studies.

**Methods:**

A total of 1125 radiomic features were extracted from clinical mammography images acquired using two different digital breast imaging systems. Feature selection was performed to reduce dimensionality and retain the most informative attributes. These were used to train a Multilayer Perceptron (MLP) regression model to estimate detectability values as defined by a model observer (MO). To increase the variability of the data and evaluate model robustness, two types of image degradation were synthetically applied to the dataset, one associated with noise and the other with blurring. The trained model was assessed using quantitative and statistical analyses, focusing on the correlation between predicted and reference detectability scores.

**Results:**

The proposed MLP regression model showed a strong correlation with the detectability values provided by the MO. Even with the introduction of degradations, the model maintained high predictive accuracy, achieving a correlation coefficient greater than 0.9.

**Conclusions:**

The combination of radiomic features and a machine learning regression model demonstrated the ability to account for variations in acquisition systems and simulated image degradations. This approach offers a promising tool for image quality assessment in clinical mammography, particularly in tasks involving microcalcification detection, without the need for labor‐intensive observer studies.

## 1 INTRODUCTION

According to the most recent data, breast cancer is among the leading causes of cancer mortality among women, with an estimated total of 43,170 deaths in 2023.^1^ However, the early detection of the disease has been shown to significantly improve treatment outcomes, as small, localized tumors are more easily treated with a higher success rate. The most reliable strategy for early diagnosis is regular screening exams, which allow for timely medical interventions.^2^ Recent research in Europe and the United States has demonstrated the efficacy of breast cancer screening programs, indicating a mortality reduction of up to 30% among women participating in these initiatives.^3^, ^4^ These findings corroborate the crucial role of mammography in reducing breast cancer deaths and enhancing long‐term survival.

In recent years, digital breast tomosynthesis (DBT) has become increasingly common in breast cancer screening. However, its availability remains limited, as the higher cost of DBT is not always fully covered by health insurance plans.^2^ Although recent studies have highlighted DBT as a promising modality,^5^, ^6^, ^7^ 2D mammography continues to be widely used in large‐scale population screening programs.^8^ Moreover, there is still no clear consensus on whether DBT offers superior performance in early cancer detection or in reducing false positives.^9^ Additionally, DBT requires longer reading times^10^ and may expose patients to higher radiation doses compared to 2D mammography.^8^, ^11^, ^12^

Regardless of the imaging modality, achieving a viable and accurate medical diagnosis requires adequate image quality, which depends on several technical factors.^13^ The applied radiation dose directly influences the signal‐to‐noise ratio (SNR), affecting the visibility of subtle structures.^14^, ^15^ Additionally, the characteristics of the digital detector and the imaging geometry of the equipment have an impact on spatial resolution, influencing the sharpness of anatomical details.^15^, ^16^ Artifacts can also compromise image quality, such as motion blur caused by patient movement during acquisition.^17^ Finally, the use of inadequate post‐processing software can lead to a reduction in image contrast, compromising the distinction between normal tissue and possible lesions.^15^

The quality of mammograms can be assessed through studies with human and/or model observers (MOs), but these approaches are complex because they require an adequate set of images, the definition of a specific clinical task, and the implementation of structured training and testing steps. In the case of studies with human observers, the cost and time required are even greater, as well as being subject to inter‐observer variability, which can affect the reproducibility of the results.^18^ Although there are traditional objective metrics for the analysis of image quality, most have been developed for natural images and may not adequately reflect the specific challenges of medical images.^18^, ^19^ In addition, full‐reference metrics require a reference image (without degradation), which is only available in simulation studies or virtual clinical trials (VCT), limiting their applicability in real clinical scenarios. Another critical point is that these metrics are quantitative and not necessarily related to a specific diagnostic task.^16^, ^20^

In practice, quality assessment methods are sought that rely solely on the acquired image itself, such as extracting features directly from the raw or processed data. Radiomics is a computational approach that extracts quantitative features from medical images using image processing and Machine Learning (ML) techniques. These features capture information that is not easily perceived by the human eye and can be converted into meaningful data to help diagnose lesions or predict relevant clinical information.^21^

Recent research has demonstrated the application of radiomics to mammograms for a variety of clinical purposes. Prinzi et al. (2024) developed a model based on features radiomics for the diagnosis of breast microcalcifications, highlighting its potential to aid in the detection and classification of these lesions.^22^ Dong et al. (2024) explored the use of explainable radiomics to characterize breast density and tissue complexity.^23^ Suzuki et al. (2023) applied radiomics to breast cancer risk prediction and demonstrated the feasibility of using these quantitative features to stratify risk.^24^ However, no studies were found that investigated the use of radiomics to predict image quality in terms of a specific task in different anatomies (thickness and density) and acquisition conditions (varying radiation dose, equipment, and presence of artifacts).

The aim of this study is to propose a method for predicting the quality of clinical digital mammography images in terms of microcalcification detectability, without relying on routine studies involving human or MOs. To achieve this, radiomic features are extracted from clinical mammograms enabling quantitative analysis of image characteristics. These features are then correlated with microcalcification detectability scores, allowing the training of a ML model capable of automatically predicting image quality in a task‐based approach.

## 2 MATERIALS & METHODS

**FIGURE 1:**
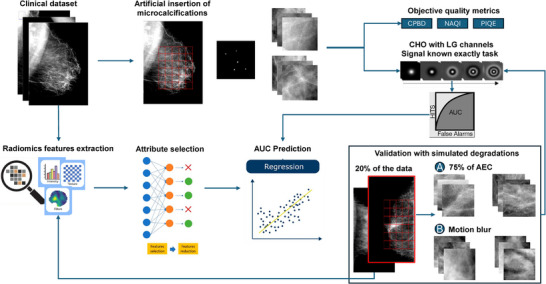
Overview of the proposal: (a) Clinical image acquisition; (b) Artificial insertion of real microcalcifications; (c) Quality assessment with objective metrics and MO; (d) Extraction and selection of radiomic features; (e) Training and testing of the regression model; and (f) Study validation with simulated degradations. MO, model observer.

Figure 1 shows the general proposal of this work, from the collection, curation, and evaluation of the clinical image database to the extraction of radiomic features for training, testing, and validation of the regression model.

### 2.1 Clinical image database

The present study uses images from two mammography units with different detector technologies, one based on a scintillator and the other on a direct detector, located in different hospitals. These two machines were chosen to analyze the impact of technology differences on the performance of the proposed model and to evaluate its ability to generalize to different acquisition systems.

From August 2021 to January 2024, 4904 clinical digital mammograms acquired on the GE Senographe Pristina 3D Mammography System (GE Healthcare, Chicago, IL, USA) were collected at the Barretos Cancer Hospital, Pio XII Foundation (Barretos, Brazil) to create our dataset #1, after approval by the Institutional Research Ethics Committee (CAAE #78625417.1.1001.5437).

Our dataset #2 was generated from September 2020 to October 2021 and consisted of 4503 clinical digital mammograms acquired on the Hologic Selenia Dimensions Mammography System (Hologic, Bedford, MA, USA), collected at the Institute of Radiology (InRad) of Hospital das Clínicas, University of São Paulo (São Paulo, Brazil) and properly submitted to the approval of the Ethics Committee (CAAE #56699016.7.0000.0065).

The acquisition parameters of both the databases are summarized in Table 1.

**TABLE 1:** Image acquisition parameters for the two digital mammography datasets used in this study.

Dataset	System	Anode/Filter	kVp	mAs	MGD (mGy)
#1	GE Senographe Pristina	Mo/Mo ‐ Rh/Ag	26–37	10–319	1–7
#2	Hologic Selenia Dimensions	W/Rh ‐ W/Ag	25–39	32–481	<0–9

*Note*: Shown are the anode/filter combinations and the ranges of tube voltage (kVp), tube current‐time product (mAs), and mean glandular dose (MGD, in mGy) for each imaging system.

Data were obtained retrospectively at both institutions from breast cancer screening examinations, including craniocaudal (CC) and medial lateral oblique (MLO) projections of the left and right breasts, using automatic exposure control (AEC) mode. All images were collected and stored in pairs: raw data, also known as DICOM “for processing”, and the processed file (DICOM “for presentation”). All clinical data were appropriately anonymized to preserve the patients' historical records.

The image exclusion criteria used in this study were as follows: (i) presence of artificial objects in the image, such as breast implants, piercings, and implantable devices for heart disease; (ii) very large breasts that prevented routine standard acquisition; (iii) extreme cases in which the images had excessive noise levels, very low contrast, and internal breast structures not visible;^25^ and (iv) absence of compatible image pairs (raw and processed). After applying the above criteria, a total of 4838 and 4434 images were selected for analysis from datasets #1 and #2, respectively.

### 2.2 Artificial insertion of microcalcifications

According to the work proposed by Borges et al. (2019),^26^ it is possible to computationally insert clusters of real microcalcifications into clinical images, resulting in hybrid images. In addition, according to another study by the same research group,^27^ these clusters can be generated several times from a set of real microcalcifications classified as small by experienced radiologists (140 ≤ϕ≤ 350 μm).

Among the 60 real microcalcifications provided in the previous study,^27^ we used five of them, with a size of about 200 μm, to generate a simulated cluster in a 1 ${\mathrm{cm}}^{2}$ window. The specific size of the lesions was defined according to the results of a recent study, which indicated that such structures of approximately that size are challenging in detection tasks.^28^

The insertion was performed by adjusting the attenuation values in the lesion location using the following equation:^27^

(1) $$I\left(x,y\right)=I_{0}\left(x,y\right)\cdot \left[1-\lambda \cdot M(x,y)\right]$$

where I is the final image with the inserted lesion, $I_{0}$ is the original (linearized) raw digital mammogram, λ is the desired contrast level, M is the binary mask representing the microcalcification, and (x,y) are the spatial coordinates within the lesion boundary.

The computational insertion into the raw clinical images was performed with a contrast of 8% (λ=0.08), a value that reduces the visibility of small microcalcifications in images from both GE and Hologic systems, according to our previous studies that conducted trials with human observers using a staircase method.^16^, ^20^, ^27^

In addition, for each image in the dataset, the microcalcification cluster was inserted into 200 random regions of breast tissue, carefully selected to be at least 5 mm away from the skin, chest wall, and pectoral muscle. These regions were identified based on the segmentation performed using LIBRA software, ensuring that the inserted clusters were located within relevant breast parenchyma tissue for subsequent acquisition of regions of interest (ROI).

### 2.3 Image quality assessment

The image database for quality assessment was generated from ROIs extracted from the raw clinical images, since the scope of this work includes a Signal‐Known‐Exactly (SKE) and Background‐Known‐Statistically (BKS) task, that is, the signal present in the analyzed region is known, while the background is statistically modeled. Accordingly, 400 ROIs of 10 ${\mathrm{mm}}^{2}$ (100x100 pixels for dataset #1 and 144x144 pixels for dataset #2) were extracted from each clinical image, equally divided into the subgroups with and without lesions, creating a total of 1,935,200 ROIs for dataset #1 and 1,773,600 ROIs for dataset #2.

#### 2.3.1 Model observer

The detection of clusters of microcalcifications in each image of the dataset was estimated using a Receiver Operating Characteristic (ROC) study with a MO, which can simulate human visual response when performing signal detection tasks. From the ROC analysis, the Area Under the ROC Curve (AUC) was used as the figure of merit.

The channelized hotelling observer (CHO), available in the VICTRE package,^29^ was used as the MO in this work. The CHO is a type of computational observer often used in the literature because it can incorporate frequency‐ or orientation‐selective channels that approximate the characteristics of the human visual system.^30^ To tune the performance of the virtual observer to match the result of human observers identifying the clusters in the previously estimated contrast,^16^, ^20^, ^27^, ^28^ five convolutional Laguerre‐Gauss (LG) channels were used, with a Gaussian width of 1.5 pixels, which corresponds approximately to the size of the microcalcifications.^31^ In addition, internal noise (σ=4.6) was added to the final variable to degrade observer performance, similar to the study by Petrov et al. (2018),^30^ so that the average detectability achieved 75%–80%, similar to our previous studies with human observers.^16^, ^20^, ^27^

In the training step, 1500 ROIs of negative cases (signal absent) and 500 ROIs where the signal was present (simulated cluster) were randomly selected from the total number of available ROIs to form the observer template based on the average signal. The larger number of negative ROIs was necessary due to the substantial variability present in clinical image patches compared to virtual images, allowing the virtual observer to robustly learn the statistical properties of the background. Fewer positive ROIs were sufficient, as they primarily define the signal template.

Regarding the test, the response given by the scalar test statistic was calculated on the 400 ROIs of each mammogram, equally divided between the two groups (signal present and signal absent). We repeated this procedure 30 times, that is, different training and test sets representing different virtual readers, to validate the consistency of the trained model by analyzing the variation of performance on each clinical image. Finally, with the score distributions, each clinical image in the dataset acquired an average AUC to describe the detectability of the microcalcification cluster.

#### 2.3.2 No‐reference objective metrics

Objective metrics commonly used in the literature were used to evaluate clinical images. Since full reference metrics require a ground truth image for comparison, which is only possible in studies with phantoms,^16^, ^20^ in this work we calculated three no‐reference metrics, as follows: NAQI (Normalized Anisotropic Quality Index),^32^ PIQE (Perception based Image Quality Evaluator),^33^ and CPBD (Cumulative Probability of Blur Detection).^34^ Although higher NAQI and CPBD values indicate better image quality, lower PIQE values indicate better perceptual quality. All metrics were estimated on the ROIs of the raw images described above, that is, without any image processing. The metric results were calculated from the mean and standard deviation of the ROI set evaluated for each clinical image.

### 2.4 Extraction and selection of radiomic features

Using the open‐source PyRadiomics library,^35^ a total of 1125 features were extracted from the clinical images. These features were obtained by applying a combination of nine image filters – original, wavelet, Laplacian of gaussian (log), square, square root, logarithm, exponential, gradient, and local binary pattern 2D (LBP) – and 7 feature classes: shape, first‐order, gray level co‐occurrence matrix (GLCM), gray level run length matrix (GLRLM), gray level size zone matrix (GLSZM), gray level dependence matrix (GLDM), and neighboring gray tone difference matrix (NGTDM). The extracted features captured details such as texture, contrast, sharpness, shape, morphology, intensity, and first‐order statistics. Features were extracted from the raw clinical images, excluding only the background (air) by applying a binary mask generated from the corresponding processed images, where the background pixels are zero or close to zero.

Feature extraction was performed using a combination of default and custom PyRadiomics parameters. Gray level discretization was set with a fixed bin width of 25, no resampling was applied to the images, and B‐spline interpolation was used when needed. Laplacian of Gaussian filtered images were generated with sigma values of 2, 3, and 4 to capture image details at different spatial scales. All other settings followed PyRadiomics default configurations. After extraction, all features were normalized using Min‐Max Scaling.

To ensure robust feature selection and avoid data leakage, a nested cross‐validation (CV) approach was adopted, combining a 10‐fold outer loop with a 5‐fold inner loop. The dataset was first randomly partitioned into ten outer folds of approximately equal size. For each outer iteration, one fold was reserved for testing, while the remaining nine were used for training. Within each training partition, a 5‐fold internal CV was conducted to perform attribute selection using a supervised feature selection algorithm. This inner loop ensured that feature selection was performed independently of the test data in each outer fold. After completing all outer iterations, the selected subsets of attributes obtained from the inner folds were aggregated, and only those features that appeared in at least 50% of the 50 inner CV folds were retained for further analysis.

Once the radiomic features were extracted and the nested CV framework established, the Correlation‐based Feature Subset Selection Evaluator (CfsSubsetEval) was applied to reduce the dimensionality of the data using the WEKA 3.8.6 toolbox.^36^ This method evaluates subsets of attributes based on the premise that good feature subsets contain variables that are highly correlated with the target variable but minimally correlated with each other.^37^ In other words, the algorithm favors attributes that provide new and relevant information for the regression task, while avoiding redundant inputs. The internal evaluation metric, known as “merit”, reflects the balance between relevance (correlation with the target) and redundancy (correlation among features). The higher the merit value (which ranges from 0 to 1), the more informative and compact the feature subset is.

To search for the optimal subset, the CfsSubsetEval method was combined with the BestFirst search strategy, configured in forward selection mode. This heuristic begins with an empty feature set and progressively adds attributes one by one. At each step, the algorithm evaluates whether the newly added attribute improves the overall merit. If no improvement is observed after several additions, the search terminates, and the subset with the highest merit score is retained. Depending on the number of initial features, tens of thousands of feature combinations may be explored, resulting in a compact and highly informative set of radiomic descriptors.

### 2.5 Prediction with machine learning

The Multilayer Perceptron (MLP) is an artificial neural network widely used for regression tasks due to its ability to model complex relationships between variables.^38^ Using WEKA, we implemented a backpropagation MLP with gradient descent to predict the continuous variable, that is, the detectability of microcalcification clusters (AUC) in each clinical image described by the MO, based on the set of features extracted and selected in the previous step.

The MLP architecture that was proposed for the regression task consisted of an input layer, three hidden layers, and an output layer. The input layer consisted of a number of neurons equal to the number of features in the dataset, and all input attributes were normalized to ensure that they were on the same scale, improving training efficiency. The first hidden layer, responsible for capturing initial and complex patterns, had 30 neurons and used the Rectified Linear Unit (ReLU) activation function. The second hidden layer, needed to refine the representations learned in the first layer and to avoid overfitting, consisted of 15 neurons, also with ReLU activation, while the third hidden layer contained five neurons, keeping the same activation function for additional compression of the extracted features. The output layer consisted of a single neuron with linear activation, suitable for predicting continuous values (regression output).

The training parameters for regularization included a learning rate and momentum of 0.05 and 1200 training epochs with a batch size of 100. The mean squared error (MSE) was used as the loss function. This setup was designed to capture more subtle interactions in the data and to efficiently update the network weights during the learning process.

Model performance was evaluated using several statistical metrics that provide a comprehensive analysis of the network's predictive ability. The correlation coefficient (r) was used to measure the strength of the linear relationship between predicted and actual values, indicating the quality of the model fit. The mean absolute error (MAE) and the root mean squared error (RMSE) were used to quantify the magnitude of the errors, with the RMSE penalizing larger errors more severely due to its quadratic formulation. In addition, the relative absolute error (RAE) and the root relative squared error (RRSE), which normalize the absolute and squared errors with respect to a reference model, were calculated to allow a relative comparison of the model's performance with respect to a base model. These metrics provided a balanced view of the MLP's performance, taking into account both the accuracy and stability of the predictions.

To assess the predictive performance of the MLP regression model, the outer folds of the nested cross‐validation scheme were used. In each of the 10 outer iterations, the model was trained using the training subset with only the features previously selected through consensus (i.e., those retained in at least 50% of the inner folds). The trained model was then evaluated on the corresponding outer test fold. This approach ensures that both feature selection and model evaluation remain strictly separated, avoiding data leakage. The final performance was calculated as the average across the ten outer folds, providing a robust estimate of the model's generalization ability and mitigating the risk of overfitting, particularly important in datasets with limited sample sizes.

### 2.6 Proposal validation

To ensure a robust and reliable model, our study included a validation section that considered adverse conditions in clinical image acquisition. Two possible degradations that could affect image quality were simulated: a reduction in radiation dose level, which increases the amount of noise and may affect the visibility of subtle structures such as microcalcifications, and the motion blur effect, which occurs due to patient movement during exposure, resulting in image blur. These conditions were included in the dataset to assess the model's ability to maintain stable performance even in challenging situations, ensuring its applicability in real‐world clinical settings.

Thus, 10% of the total clinical images from each dataset were randomly selected for dose reduction simulation using a specific method developed for this purpose.^39^, ^40^ The noise injection method aimed to simulate acquisition conditions at 75% of the AEC, resulting in a reduction in SNR. In addition, another 10% of the images were randomly selected to simulate the motion blur effect, reproducing the effect of the patient's movement during acquisition. This degradation was applied by convolving the original images with a 5x5 pixel directional blur kernel with an angle of 45 degrees, representing possible movements in clinical examinations.

**FIGURE 2:**
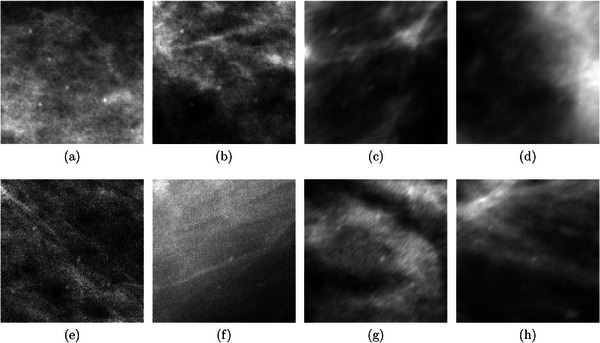
Validation ROIs: (a‐b) 75% of the AEC and (c‐d) Motion blur from dataset #1; (e‐f) 75% of the AEC and (g‐h) Motion blur from dataset #2. AEC, automatic exposure control; ROI, regions of interest.

Figure 2 shows examples of ROIs from datasets #1 and #2 in which the clustered microcalcifications were computationally inserted and then simulated degradation was added, resulting in increased visible noise and blur.

Similarly to the procedure applied to the clinical images acquired in AEC mode, the extraction of radiomic features was performed both on the set of images with reduced radiation dose and on the set affected by motion blur, with the purpose of retraining and testing the regression model. In these scenarios, a modified feature selection threshold was adopted: instead of retaining attributes present in at least 50% of the inner folds, features selected in at least 10% of the 50 inner folds were kept. This adjustment was necessary to account for the limited representation of each degradation type, which affected only 10% of the images in the dataset. Lowering the consensus threshold allowed the inclusion of features that may be specifically informative for the altered imaging conditions. This strategy ensures that the model is exposed to degradation‐specific patterns during training, improving its ability to generalize and maintain performance in the presence of such adverse conditions.

In line with the initial setup, the ROIs extracted from the sets were evaluated by the same MO, following the previously described task, in order to provide the target (detection) values for the training and evaluation of the final prediction model. The settings, training and testing protocols of the MO were kept identical for the images generated in this experiment.

## 3 RESULTS

**FIGURE 3:**
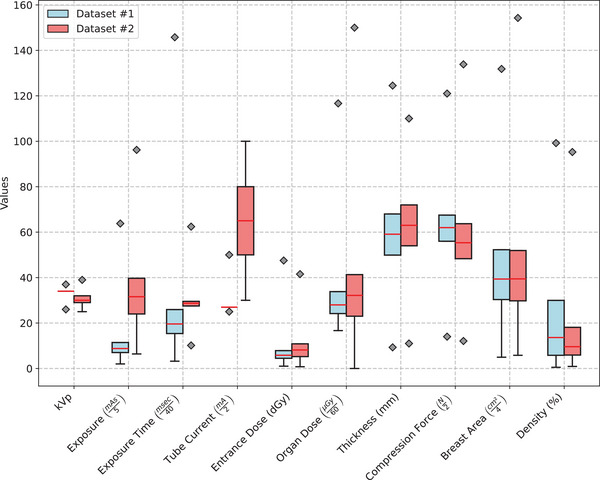
DICOM header and LIBRA data extracted from datasets #1 and #2. LIBRA, laboratory for individualized breast radiodensity assessment.

Figure 3 shows the statistical analysis of the distinct values of kVp, mAs, compressed breast thickness (CBT), and other data provided in the DICOM header of the clinical images from datasets #1 and #2. In addition, the area and percentage of breast density were extracted from the processed images using the Laboratory for Individualized Breast Radiodensity Assessment (LIBRA) tool^41^ and included in the population analysis.

As we can see in Figure 3, both datasets showed a high variability in anatomy, reflecting the diversity of mammographic features present in the images, as well as differences in dose‐related acquisition parameters. However, as an exception, the acquisition parameters of the dataset #1 revealed that both the tube voltage (kVp) and the current were not as variable as in the dataset #2. The rationale behind the difference is that the GE manufacturer predominantly adopts two fixed kV points for the majority of acquisitions,^42^ resulting in less dispersion of those values across the dataset.

**FIGURE 4:**
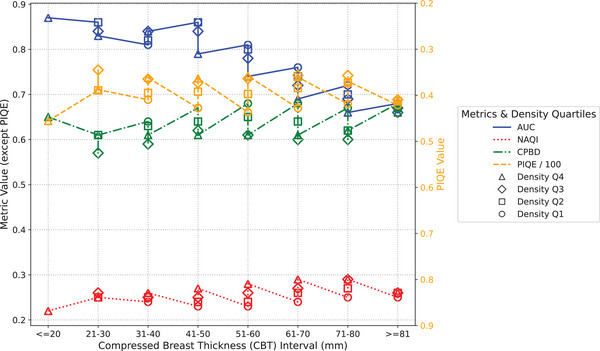
MO and no‐reference objective metrics, by CBT interval and density quartile (dataset #1). CBT, compressed breast thickness; MO, model observer.

**FIGURE 5:**
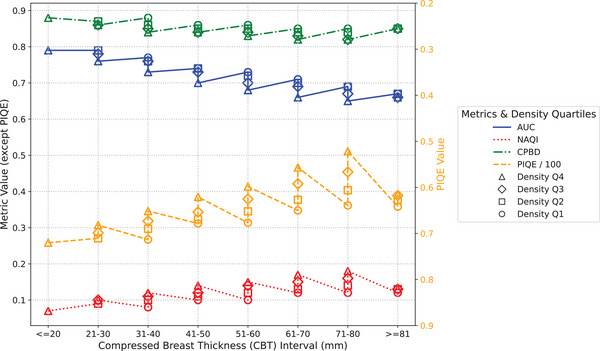
MO and no‐reference objective metrics, by CBT interval and density quartile (dataset #2). CBT, compressed breast thickness; MO, model observer.

Figures 4 and 5 show the results of both the MO and the objective metrics for the clinical images from datasets #1 and #2, respectively, acquired in AEC mode. The results were organized by CBT range and density quartile, considering the values from the DICOM header and the LIBRA automated breast density estimation software, respectively.

As we can see in both figures, the detectability of microcalcifications tends to decrease as breast thickness is increased. Another related result is that increased breast density also has a negative impact on the detectability of microcalcifications. Both results are in agreement with similar previous studies.^43^, ^44^ Nonetheless, it is important to note that these findings were not observed for dataset #1 in the CBT 31–40 range, possibly due to the change in kVp (from 26 to 34) applied by the GE machine, depending on the anatomy being imaged and the thresholds predefined by the manufacturer.^42^

In contrast, for both thickness and density variation, none of the applied no‐reference objective metrics were able to describe the reduction in quality observed by the MO. When comparing different compressed breast thickness groups within the same density quartile, the results of the CPBD and NAQI metrics showed little or no variation, especially for thicknesses closer to the population mean, while the PIQE metric showed differences between groups, although there was no agreement with the trend shown by the MO (AUC). Although the subgroups with high breast density had a negative impact on the quality of clinical images, the NAQI and PIQE metrics indicated the opposite. In the case of the CPBD metric, the results do not show convergence with the AUC values from MO.

**FIGURE 6:**
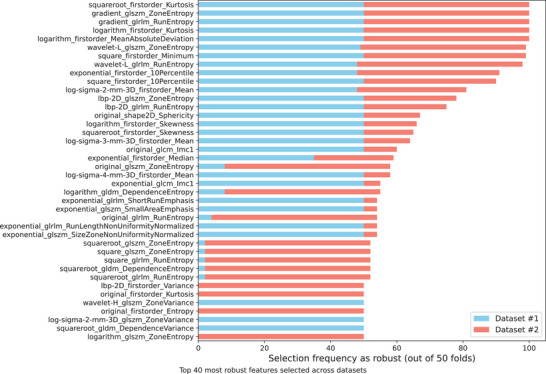
Top 40 most frequently selected features across datasets #1 and #2.

Figure 6 presents the top 40 most frequently selected radiomic features across datasets #1 and #2, based on their robustness and relevance identified through the CfsSubsetEval method within a nested cross‐validation framework (10‐fold CV with 5‐fold inner CV). The horizontal stacked bar chart illustrates the selection frequency of each feature across 50 validation folds, with features ranked by their overall frequency of selection.

It can be seen that at least two features extracted in Gradient, two in LBP (texture descriptor) and two in Wavelet‐L (low‐pass filter) were considered relevant for both datasets. For these three cases, GLSZM and GLRLM features were selected, indicating a possible relevant pattern for modeling. The GLSZM features quantify gray level zones in the image, while GLRLM quantifies the number of consecutive pixels that have the same gray level value. In addition, first‐order features were also relevant to both datasets and were selected in four different domains: logarithm, square, square root and exponential, further supporting the relevance of multi‐domain information in radiomic analysis.

Overall, 50 features were selected for dataset #1 and 54 for dataset #2 (from a total of 1125 available features), with 13 features shared by both datasets, based on a selection consensus of at least 50% of the inner CV folds (Supporting Material – Table 4). This yields an instance‐to‐feature ratio of approximately 96.8 for dataset #1 and 82.1 for dataset #2, representing a substantial improvement over the initial ratios of ∼4.3 and ∼3.9, respectively, and reflecting a reduction of over 95% in the original number of features. Such ratios ensure a more stable training configuration, reducing the risk of overfitting and improving the model's ability to learn meaningful patterns rather than noise. Moreover, the differences in the selected features between datasets highlight the influence of image acquisition methods, reinforcing the importance of treating the datasets independently rather than enforcing a unified model or feature set across both.

Table 2 shows a comparison of the performance metrics for the MLP regression model based on datasets #1 and #2. From this table, we can assess the accuracy of the model predictions relative to the actual AUC values of the MO achieved in the microcalcification detection task.

**TABLE 2:** Mean and standard deviation of performance metrics for the MLP regression model.

	Dataset #1		Dataset #2	
Metric	Mean	Std	Mean	Std
Correlation Coefficient (r)	0.9734	0.0035	0.9367	0.0085
MAE	0.0109	0.0004	0.0093	0.0004
RMSE	0.0141	0.0005	0.0121	0.0007
RAE (%)	21.68	1.35	33.37	1.86
RRSE (%)	23.29	1.41	35.14	2.18

Abbreviations: MAE, mean absolute error; MLP, multilayer perceptron; RAE, relative absolute error; RMSE, root mean square error; RRSE, root relative squared error.

The correlation coefficient (r) indicates that both datasets combined with the MLP regression model produced predicted values that were closely related to the actual ones (r>0.9), however, dataset #1 had a higher correlation index than dataset #2. Moreover, the MAE and RMSE were slightly lower for dataset #2, suggesting more stable predictions with less variation in magnitude. On the other hand, the relative errors were higher when predicting using dataset #2, indicating that the regression model may deviate more proportionally compared to the dataset #1. These findings show that the regression performance differs depending on the acquisition system, highlighting the benefit of taking these differences into account to ensure the best generalization of the results.

**FIGURE 7:**
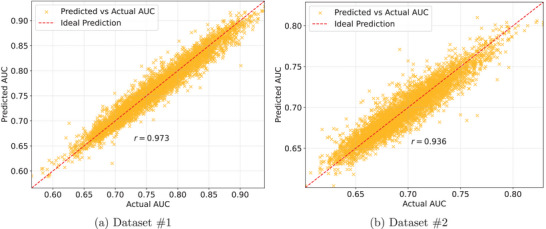
Scatter plots comparing actual AUCs with predicted ones from the regression model with (a) dataset #1 and (b) dataset #2.

Figure 7 shows the scatter plot comparing the actual AUC values with the predicted ones for each dataset. The dashed red line represents the ideal prediction, where the predicted values would be exactly the same as the actual ones.

The scatter plots show the strong linear relationship between the actual and predicted AUC values for both datasets, indicating a good performance of the model trained separately for each dataset. In the regression with dataset #1, the points were more concentrated near the ideal prediction line, an indication that the model trained for this dataset provides more accurate predictions, even with the wide AUC range. In dataset #2, the AUC range is reduced, but the relative error is slightly higher due to the more scattered points on the plot. These differences highlight the fact that although the populations are similar, there are significant variations in the data generated by each acquisition system, and consequently in the detectability and representation of radiomic features, which could affect the generalizability of the predictions if only a single regression model were used for the full set of data.

**FIGURE 8:**
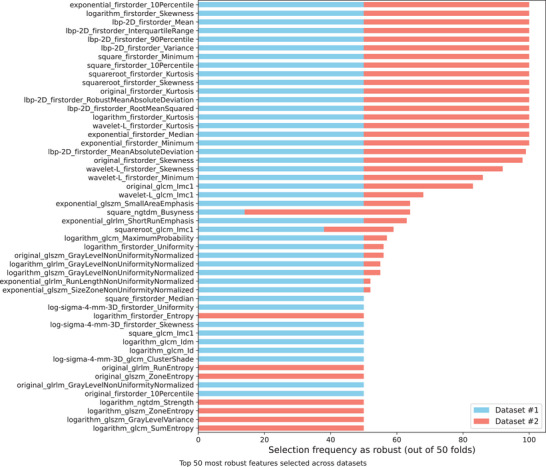
Top 50 most frequently selected features across validation datasets #1 and #2.

Finally, by increasing the data variance to more closely resemble clinical reality, our proposal and datasets were evaluated for robustness under different original clinical image degradation conditions. Figure 8 summarizes the top 50 most frequently selected features across validation datasets #1 and #2, in which 10% of the clinical images were degraded by inserting additional noise, adjusting them to 75% of the standard acquisition dose (AEC), while another 10% were modified by simulating motion blur. The horizontal stacked bar chart shows the frequency with which each feature was selected as robust across 50 nested CV folds (10‐fold CV with 5‐fold inner CV). The detailed list of features used in the validation model can be found in the supporting material of this study (Table 5).

Once again, we can see the differences in the distribution of the features selected for the validation model with datasets #1 and #2 (Figure 8). When applying the rule that a feature must be selected in at least 10% of the inner CV folds, only 32 features were commonly selected across both datasets out of the 118 and 122 features considered for datasets #1 and #2, respectively. Most selected features are first‐order statistics from transformed images and texture descriptors (GLCM, GLSZM, GLRLM, NGTDM), emphasizing kurtosis, skewness, and percentiles across multiple domains.

The difference in the number and type of features between the datasets confirmed the fact that the same set of attributes may not be equally representative for both acquisition systems, implying the need to train exclusive regression models. The choice of features may also indicate that the effect of image degradation (noise or motion blur) affects each system differently, requiring specific tuning for each dataset.

Although the features selected for each dataset were more divergent than similar, both datasets incorporated filters such as wavelet, logarithm, and LBP, indicating that the selection of attributes leveraged multiple texture scales and nonlinear transformations to improve discrimination of patterns in the images.

Table 3 compares the performance metrics of the MLP regression model in the validation stage, again trained on datasets #1 and #2.

**TABLE 3:** Performance metrics (mean and standard deviation) for the MLP regression model in the validation stage.

	Dataset #1		Dataset #2	
Metric	Mean	Std	Mean	Std
Correlation Coefficient (r)	0.9728	0.0024	0.9316	0.0107
MAE	0.0115	0.0005	0.0100	0.0006
RMSE	0.0153	0.0008	0.0134	0.0010
RAE (%)	21.81	1.13	35.27	2.78
RRSE (%)	23.51	1.40	37.57	3.14

Abbreviations: MAE, mean absolute error; MLP, multilayer perceptron; RAE, relative absolute error; RMSE, root mean square error; RRSE, root relative squared error.

The results of the validation step confirm the trends observed previously, indicating a strong correlation between the model responses and the expected values. Despite the inclusion of degradations in part of the dataset, both datasets maintained a high correlation coefficient, demonstrating that the predicted values are close to the actual AUCs, even when there are images of reduced quality. However, the relative errors remain higher for the model trained on dataset #2 compared to #1, suggesting that although the predictions are consistent, we have a greater range of deviation from the real values in dataset #2.

## 4 DISCUSSION

The aim of this study was to propose a model based on radiomics and machine learning to predict clinical image quality in terms of detectability of microcalcifications in mammograms, without the need for routine evaluation by human or MOs. Therefore, radiomic features were extracted from clinical images acquired under different conditions, including variations in radiation dose, the use of two different acquisition systems, and the presence of motion blur artifacts. These features were then correlated with the detectability of microcalcifications computed by a MO, and used to train and validate a predictive model that allows task‐based assessment of image quality in adverse conditions using only the raw acquisition data.

The results showed that breast thickness and density had a significant impact on the detectability of microcalcifications, which emphasizes the importance of those factors in the formation and quality of the acquired image; however, the no‐reference objective metrics CPBD, NAQI and PIQE were not able to show the same findings. A previous study had already demonstrated the impact of compressed breast thickness and dose on the detectability of lesions in digital mammography, especially in the case of microcalcifications, which were also computationally inserted into clinical images.^43^

In terms of breast anatomy, it is known that there is a relationship between thickness and volumetric density. Previous studies have shown that thinner breasts tend to have a higher volumetric breast density because of the higher proportion of fibroglandular tissue in relation to the total volume. On the other hand, thicker breasts may contain a greater absolute amount of fibroglandular tissue, although its proportion to total volume is lower due to the presence of more adipose tissue.^45^ This relationship is relevant because breast composition influences the attenuation of x‐rays, which affects image quality and the detectability of lesions in mammography.^44^

Regarding no‐reference objective metrics, we should mention that previous studies have already shown their limitations in assessing mammographic image quality.^16^, ^20^ Although a previous study showed that NAQI decreases with increasing breast thickness and increases with density,^32^ this study observed the same effect in relation to density, but the gradual increase in thickness did not necessarily lead to lower NAQI values. This difference may be related to the fact that the former evaluated only processed images, while the latter analyzed “for processing” images, which preserve the original acquisition data. Furthermore, many of those metrics were originally developed for natural images and do not take into account the properties of mammography, such as the low SNR and the presence of overlapping structures.^18^, ^19^

As demonstrated in this work, the radiomics features extracted from clinical images were able to capture patterns related to the visibility of microcalcifications, even reflecting variations in image quality due to different parameters and acquisition settings. The MLP regression model performed consistently well in predicting detectability values, with metrics indicating a strong correlation between the predictions and the reference AUCs, both for the original data (“no distortion applied”) and for the simulated ones included in the validation step.

Even though datasets #1 and #2 share some of the same feature classes, there are considerable differences in the final selection, which shows that both the image acquisition and the impact of the applied transformations directly influence the set of relevant attributes for modeling. This might be related to the specificities of each acquisition system, as the Hologic mammography unit uses a direct conversion detector (a‐Se) with a pixel size of 70 μm, while the GE uses a CsI(Tl) detector technology, based on indirect conversion of energy to visible light, with a pixel size of 100 μm. Such differences can impact the spatial resolution, pixel cross‐talk, and noise response,^46^ thus affecting the detectability of objects^16^ and, in our study, the selection of the most representative features for each system.

Comparing the initial step with the validation one, the latter required a larger number of features to describe each dataset. We expected that since the images have undergone transformations that include different aspects, such as increased visible noise and blurring of details (smearing), requiring a more comprehensive set of attributes to capture these variations and ensure the robustness of the prediction model.

Our study has several limitations that should be considered in future research. First, only one clinical task was evaluated, that is, the detection of microcalcifications, whereas other tasks, such as the detection and discrimination of masses, are equally relevant. In addition, only one size of microcalcifications was investigated, and it is essential to include other sizes of these lesions, since detectability depends on the size of these structures.^28^ Another limitation, common to studies using radiomics and clinical data,^23^, ^24^ relates to the number of images used to extract features and train the machine learning model, which could be increased to cover a greater anatomical diversity and thus reduce the prediction error. Furthermore, only two types of degradation were simulated in this study—one associated with noise and the other with blur—but there are other sources of degradation specific to mammography^15^ that could be explored to assess whether the model would produce consistent results in a wider range of settings.

In addition, to avoid any influence and/or dependence on the post‐processing algorithm, all experiments were conducted on “for‐processing” images only, that is, without any additional image processing by the equipment's software. In other words, processed images were not evaluated, which could have a direct impact on the applicability of the ML model in clinical workflows that only provide images that have already been processed for radiological interpretation.

Another limitation of this study is that the analysis focused exclusively on the individual behavior of radiomic features under scenarios of original acquisitions (as defined by the equipment settings) and simulated degradation through reduced SNR and image blurring. Although this approach is essential for understanding the stability of features under varying image quality conditions, it does not include the evaluation of composite radiomic signatures, defined as combinations of features obtained through clustering techniques, dimensionality reduction, or multivariate modeling. The construction and validation of such signatures would require specific methodologies beyond the scope of this work, but represent a relevant direction for future studies aimed at the clinical application of more robust predictive models.

Finally, a fundamental premise for the viability of this proposal is to predetermine the detectability values in the clinical task, which in our case was done with a MO due to the large number of images to be scored. Alternatively, those values could be provided once by human observers, based on subsets of images under different conditions. After this first stage of task‐based performance data collection, no further observer studies are required because the prediction model has already been tuned and automated, allowing continuous assessment of image quality objectively and independently of human intervention.

## 5 CONCLUSIONS

Given the limitations of traditional objective metrics, the radiomics and ML approach used in this study is a promising alternative for predicting image quality. Although full‐reference metrics rely on reference images, which are not available for clinical imaging, and no‐reference metrics have proven to be inadequate for evaluating this type of data, our proposal has been demonstrated to be suitable for describing image quality in terms of detectability of microcalcifications. In addition, the trained model was able to address variations imposed by different acquisition systems and introduced degradations such as noise and blur, demonstrating its potential for application in real‐world clinical practice.

Although observer studies are desirable for a more robust validation of visual perception of image quality, their implementation is costly and time‐consuming, and hence, automated approaches, such as the one proposed in this paper, are a feasible and powerful alternative. Moreover, the application of this method requires no external software and is not dependent on image processing, relying exclusively on the extraction of features from the image obtained directly from the acquisition system. Future work may explore the development and validation of radiomic signatures, to assess whether combinations of stable features can maintain predictive robustness under image degradation scenarios.

## CONFLICT OF INTEREST STATEMENT

The authors declare no conflicts of interest.

## Supporting information

Supporting Material
