# Supplementary material for: Predicting the quality of digital mammography from the perspective of detectability of microcalcifications: A Radiomics approach
Source: Med Phys. 2025 Aug 15;52(8):e18061. doi: 10.1002/mp.18061 (PMC12356991; doi:10.1002/mp.18061)
Supplement: Supplementary file 1 — Supporting Material [file MP-52-0-s001.pdf]

Supporting Material

The following tables provide the full list of radiomics features extracted and selected to train the MLP regression model. These features cover a variety of pre-processing filters and feature types, and were obtained from images acquired on datasets #1 and #2.

Table 4: Features selected for initial model

Feature	Dataset #1	Dataset #2
exponential_firstorder_10Percentile	✓	✓
exponential_firstorder_Median	✓	
exponential_glcm_Imc1	✓	
exponential_glrlm_RunLengthNonUniformityNormalized	✓	
exponential_glrlm_ShortRunEmphasis	✓	
exponential_glszm_SizeZoneNonUniformityNormalized	✓	
exponential_glszm_SmallAreaEmphasis	✓	
gradient_glcm_Correlation	✓	
gradient_glcm_MCC	✓	
gradient_glrlm_RunEntropy	✓	✓
gradient_glszm_ZoneEntropy	✓	✓
lbp-2D_firstorder_MeanAbsoluteDeviation		✓
lbp-2D_firstorder_Median		✓
lbp-2D_firstorder_Variance		✓
lbp-2D_glrlm_RunEntropy	✓	✓
lbp-2D_glrlm_ShortRunEmphasis	✓	
lbp-2D_glrlm_ShortRunHighGrayLevelEmphasis	✓	
lbp-2D_glrlm_ShortRunLowGrayLevelEmphasis	✓	
lbp-2D_glszm_SmallAreaEmphasis	✓	
lbp-2D_glszm_SmallAreaHighGrayLevelEmphasis	✓	
lbp-2D_glszm_SmallAreaLowGrayLevelEmphasis	✓	
lbp-2D_glszm_ZoneEntropy	✓	✓
log-sigma-2-mm-3D_firstorder_Kurtosis	✓	
log-sigma-2-mm-3D_firstorder_Mean	✓	✓
log-sigma-2-mm-3D_firstorder_MeanAbsoluteDeviation		✓
log-sigma-2-mm-3D_firstorder_Skewness	✓	
log-sigma-2-mm-3D_glrlm_RunVariance	✓	
log-sigma-2-mm-3D_glszm_LargeAreaLowGrayLevelEmphasis		✓
log-sigma-2-mm-3D_glszm_ZoneVariance	✓	
log-sigma-3-mm-3D_firstorder_Mean	✓	
log-sigma-3-mm-3D_firstorder_Median		✓
log-sigma-3-mm-3D_firstorder_Skewness	✓	

Continue on the next page...

Continuation of Table 4

Feature	Dataset #1	Dataset #2
log-sigma-3-mm-3D_glszm_LargeAreaLowGrayLevelEmphasis		✓
log-sigma-4-mm-3D_firstorder_10Percentile		✓
log-sigma-4-mm-3D_firstorder_Mean	✓	
log-sigma-4-mm-3D_firstorder_Median		✓
log-sigma-4-mm-3D_firstorder_Skewness	✓	
log-sigma-4-mm-3D_glcm_ClusterTendency	✓	✓
log-sigma-4-mm-3D_gldm_DependenceEntropy		✓
log-sigma-4-mm-3D_glrlm_LongRunLowGrayLevelEmphasis		✓
log-sigma-4-mm-3D_glszm_LargeAreaLowGrayLevelEmphasis		✓
logarithm_firstorder_Kurtosis	✓	✓
logarithm_firstorder_MeanAbsoluteDeviation	✓	✓
logarithm_firstorder_RobustMeanAbsoluteDeviation		✓
logarithm_firstorder_Skewness	✓	
logarithm_glcm_SumEntropy		✓
logarithm_gldm_DependenceEntropy		✓
logarithm_glrlm_RunEntropy		✓
logarithm_glszm_ZoneEntropy		✓
original_firstorder_Entropy		✓
original_firstorder_Kurtosis		✓
original_glcm_Imc1	✓	
original_glcm_SumEntropy		✓
original_gldm_DependenceEntropy		✓
original_glrlm_LongRunLowGrayLevelEmphasis	✓	
original_glrlm_RunEntropy		✓
original_glszm_LargeAreaLowGrayLevelEmphasis	✓	
original_glszm_ZoneEntropy		✓
original_ngtdm_Busyness		✓
original_shape2D_Sphericity	✓	
square_firstorder_10Percentile	✓	✓
square_firstorder_Minimum	✓	✓
square_gldm_LargeDependenceLowGrayLevelEmphasis	✓	
square_gldm_LowGrayLevelEmphasis	✓	
square_glrlm_RunEntropy		✓
square_glszm_ZoneEntropy		✓
square_ngtdm_Busyness		✓
squareroot_firstorder_Entropy		✓
squareroot_firstorder_Kurtosis	✓	✓
squareroot_firstorder_MeanAbsoluteDeviation		✓
squareroot_firstorder_Skewness	✓	
squareroot_gldm_DependenceEntropy		✓

Continue on the next page...

Continuation of Table 4

Feature	Dataset #1	Dataset #2
squareroot_gldm_DependenceVariance	✓	
squareroot_glcm_SumEntropy		✓
squareroot_glrlm_RunEntropy		✓
squareroot_glszm_ZoneEntropy		✓
wavelet-H_firstorder_Skewness		✓
wavelet-H_glcm_ClusterTendency	✓	
wavelet-H_glcm_Correlation	✓	
wavelet-H_gldm_LargeDependenceEmphasis	✓	
wavelet-H_glrlm_RunVariance	✓	
wavelet-H_glszm_ZoneVariance	✓	
wavelet-L_firstorder_Entropy		✓
wavelet-L_firstorder_Kurtosis		✓
wavelet-L_glcm_JointEntropy		✓
wavelet-L_glcm_SumEntropy		✓
wavelet-L_gldm_DependenceEntropy		✓
wavelet-L_glrlm_RunEntropy	✓	✓
wavelet-L_glszm_ZoneEntropy	✓	✓
wavelet-L_ngtdm_Busyness		✓

Table 5: Features selected for validation model

Feature	Dataset #1	Dataset #2
exponential_firstorder_10Percentile	✓	✓
exponential_firstorder_Median	✓	✓
exponential_firstorder_Minimum	✓	✓
exponential_firstorder_Range		✓
exponential_firstorder_Uniformity	✓	
exponential_glcm_Imc1	✓	
exponential_glcm_JointEnergy	✓	
exponential_glcm_MCC		✓
exponential_glcm_MaximumProbability	✓	
exponential_gldm_LargeDependenceEmphasis		✓
exponential_gldm_LargeDependenceLowGrayLevelEmphasis	✓	
exponential_gldm_LowGrayLevelEmphasis	✓	
exponential_gldm_SmallDependenceLowGrayLevelEmphasis	✓	
exponential_glrlm_GrayLevelVariance		✓
exponential_glrlm_RunEntropy		✓
exponential_glrlm_RunLengthNonUniformityNormalized	✓	
exponential_glrlm_ShortRunEmphasis	✓	✓

Continue on the next page...

Continuation of Table 5

Feature	Dataset #1	Dataset #2
exponential_glszm_GrayLevelVariance		✓
exponential_glszm_SizeZoneNonUniformityNormalized	✓	
exponential_glszm_SmallAreaEmphasis	✓	✓
exponential_glszm_ZoneEntropy		✓
exponential_ngtdm_Busyness	✓	
exponential_ngtdm_Coarseness	✓	
exponential_ngtdm_Complexity		✓
exponential_ngtdm_Strength		✓
gradient_firstorder_10Percentile	✓	
gradient_firstorder_Uniformity	✓	
gradient_glcm_Correlation		✓
gradient_glcm_Imc1	✓	
gradient_glcm_Imc2		✓
gradient_glcm_JointEnergy	✓	
gradient_glcm_MaximumProbability	✓	
gradient_glszm_GrayLevelNonUniformityNormalized	✓	
gradient_glszm_LowGrayLevelZoneEmphasis	✓	
gradient_glszm_SmallAreaHighGrayLevelEmphasis		✓
gradient_glszm_SmallAreaLowGrayLevelEmphasis	✓	
gradient_gldm_LargeDependenceLowGrayLevelEmphasis	✓	
gradient_gldm_LowGrayLevelEmphasis	✓	
gradient_gldm_SmallDependenceLowGrayLevelEmphasis	✓	
gradient_glrlm_GrayLevelNonUniformityNormalized	✓	
gradient_glrlm_LowGrayLevelRunEmphasis	✓	
gradient_glrlm_ShortRunLowGrayLevelEmphasis	✓	
lbp-2D_firstorder_10Percentile		✓
lbp-2D_firstorder_90Percentile	✓	✓
lbp-2D_firstorder_InterquartileRange	✓	✓
lbp-2D_firstorder_Kurtosis		✓
lbp-2D_firstorder_Mean	✓	✓
lbp-2D_firstorder_MeanAbsoluteDeviation	✓	✓
lbp-2D_firstorder_Median		✓
lbp-2D_firstorder_RobustMeanAbsoluteDeviation	✓	✓
lbp-2D_firstorder_RootMeanSquared	✓	✓
lbp-2D_firstorder_Skewness		✓
lbp-2D_firstorder_Variance	✓	✓
lbp-2D_glrlm_RunEntropy		✓
lbp-2D_glrlm_RunLengthNonUniformityNormalized		✓
lbp-2D_glrlm_ShortRunEmphasis		✓
lbp-2D_glrlm_ShortRunHighGrayLevelEmphasis		✓

Continue on the next page...

Continuation of Table 5

Feature	Dataset #1	Dataset #2
lbp-2D_glrlm_ShortRunLowGrayLevelEmphasis		✓
lbp-2D_glszm_SizeZoneNonUniformityNormalized		✓
lbp-2D_glszm_SmallAreaEmphasis		✓
lbp-2D_glszm_SmallAreaHighGrayLevelEmphasis		✓
lbp-2D_glszm_SmallAreaLowGrayLevelEmphasis		✓
lbp-2D_glszm_ZoneEntropy		✓
log-sigma-2-mm-3D_firstorder_Mean	✓	
log-sigma-2-mm-3D_firstorder_Minimum	✓	
log-sigma-2-mm-3D_firstorder_Skewness	✓	
log-sigma-2-mm-3D_firstorder_Uniformity	✓	
log-sigma-2-mm-3D_glcm_JointEnergy	✓	
log-sigma-2-mm-3D_glcm_MaximumProbability	✓	
log-sigma-2-mm-3D_glrlm_GrayLevelNonUniformityNormalized	✓	
log-sigma-2-mm-3D_glszm_GrayLevelNonUniformityNormalized	✓	
log-sigma-2-mm-3D_ngtdm_Contrast	✓	
log-sigma-3-mm-3D_firstorder_Mean	✓	
log-sigma-3-mm-3D_firstorder_Minimum	✓	
log-sigma-3-mm-3D_firstorder_Skewness	✓	
log-sigma-3-mm-3D_firstorder_Uniformity	✓	
log-sigma-3-mm-3D_glcm_ClusterShade	✓	
log-sigma-3-mm-3D_glcm_JointEnergy	✓	
log-sigma-3-mm-3D_glcm_MaximumProbability	✓	
log-sigma-3-mm-3D_glrlm_GrayLevelNonUniformityNormalized	✓	
log-sigma-3-mm-3D_glszm_GrayLevelNonUniformityNormalized	✓	
log-sigma-3-mm-3D_glszm_LargeAreaLowGrayLevelEmphasis		✓
log-sigma-3-mm-3D_glszm_SmallAreaLowGrayLevelEmphasis	✓	
log-sigma-4-mm-3D_firstorder_Mean	✓	
log-sigma-4-mm-3D_firstorder_Minimum	✓	
log-sigma-4-mm-3D_firstorder_Skewness	✓	
log-sigma-4-mm-3D_firstorder_Uniformity	✓	
log-sigma-4-mm-3D_glcm_ClusterShade	✓	
log-sigma-4-mm-3D_glcm_JointEnergy	✓	
log-sigma-4-mm-3D_glcm_MaximumProbability	✓	
log-sigma-4-mm-3D_glrlm_LongRunEmphasis		✓
log-sigma-4-mm-3D_glrlm_LongRunLowGrayLevelEmphasis		✓
log-sigma-4-mm-3D_glszm_LargeAreaEmphasis		✓
log-sigma-4-mm-3D_glszm_LargeAreaLowGrayLevelEmphasis		✓
log-sigma-4-mm-3D_ngtdm_Busyness		✓
logarithm_firstorder_Entropy		✓
logarithm_firstorder_InterquartileRange		✓

Continue on the next page...

Continuation of Table 5

Feature	Dataset #1	Dataset #2
logarithm_firstorder_Kurtosis	✓	✓
logarithm_firstorder_MeanAbsoluteDeviation		✓
logarithm_firstorder_RobustMeanAbsoluteDeviation		✓
logarithm_firstorder_Skewness	✓	✓
logarithm_firstorder_Uniformity	✓	✓
logarithm_glcm_Id	✓	
logarithm_glcm_Idm	✓	
logarithm_glcm_Imc1		✓
logarithm_glcm_JointEnergy	✓	
logarithm_glcm_MaximumProbability	✓	✓
logarithm_glcm_SumEntropy		✓
logarithm_glszm_GrayLevelNonUniformityNormalized	✓	✓
logarithm_glszm_GrayLevelVariance		✓
logarithm_glszm_LargeAreaEmphasis		✓
logarithm_glszm_LargeAreaLowGrayLevelEmphasis		✓
logarithm_glszm_ZoneEntropy		✓
logarithm_glszm_ZoneVariance		✓
logarithm_gldm_DependenceEntropy		✓
logarithm_gldm_GrayLevelNonUniformity	✓	
logarithm_gldm_LargeDependenceEmphasis	✓	
logarithm_glrlm_GrayLevelNonUniformityNormalized	✓	✓
logarithm_glrlm_LongRunLowGrayLevelEmphasis		✓
logarithm_glrlm_RunEntropy		✓
logarithm_glrlm_RunVariance		✓
logarithm_ngtdm_Strength		✓
original_firstorder_10Percentile	✓	
original_firstorder_Entropy		✓
original_firstorder_Kurtosis	✓	✓
original_firstorder_RobustMeanAbsoluteDeviation		✓
original_firstorder_Skewness	✓	✓
original_firstorder_Uniformity		✓
original_glcm_Imc1	✓	✓
original_glcm_InverseVariance	✓	
original_glcm_SumEntropy		✓
original_glrlm_GrayLevelNonUniformityNormalized	✓	
original_glrlm_RunEntropy		✓
original_glszm_GrayLevelNonUniformityNormalized	✓	✓
original_glszm_ZoneEntropy		✓
original_ngtdm_Strength		✓
square_firstorder_10Percentile	✓	✓

Continue on the next page...

Continuation of Table 5

Feature	Dataset #1	Dataset #2
square_firstorder_Kurtosis		✓
square_firstorder_Median	✓	
square_firstorder_Minimum	✓	✓
square_firstorder_Skewness		✓
square_glcm_Imc1	✓	
square_glrlm_GrayLevelNonUniformity	✓	
square_glrlm_GrayLevelNonUniformityNormalized	✓	
square_glrlm_RunEntropy		✓
square_glszm_GrayLevelNonUniformity	✓	
square_glszm_GrayLevelNonUniformityNormalized	✓	
square_glszm_ZoneEntropy		✓
square_ngtdm_Busyness	✓	✓
square_ngtdm_Strength		✓
squareroot_firstorder_Entropy		✓
squareroot_firstorder_Kurtosis	✓	✓
squareroot_firstorder_MeanAbsoluteDeviation		✓
squareroot_firstorder_RobustMeanAbsoluteDeviation		✓
squareroot_firstorder_Skewness	✓	✓
squareroot_firstorder_Uniformity	✓	✓
squareroot_glcm_Imc1	✓	✓
squareroot_glcm_JointEnergy	✓	
squareroot_glcm_MaximumProbability	✓	
squareroot_glcm_SumEntropy		✓
squareroot_gldm_DependenceEntropy		✓
squareroot_glrlm_GrayLevelNonUniformityNormalized	✓	
squareroot_glrlm_LongRunLowGrayLevelEmphasis		✓
squareroot_glrlm_RunEntropy		✓
squareroot_glrlm_RunVariance		✓
squareroot_glszm_GrayLevelNonUniformityNormalized	✓	
squareroot_glszm_LargeAreaLowGrayLevelEmphasis		✓
squareroot_glszm_ZoneEntropy		✓
squareroot_glszm_ZoneVariance		✓
squareroot_ngtdm_Busyness		✓
squareroot_ngtdm_Strength		✓
wavelet-H_firstorder_InterquartileRange	✓	
wavelet-H_firstorder_Minimum	✓	
wavelet-H_firstorder_RobustMeanAbsoluteDeviation	✓	
wavelet-H_firstorder_Skewness		✓
wavelet-H_glcm_Correlation		✓
wavelet-H_glcm_InverseVariance	✓	

Continue on the next page...

Continuation of Table 5

Feature	Dataset #1	Dataset #2
wavelet-H_glrlm_GrayLevelNonUniformityNormalized	✓	
wavelet-H_glrlm_LongRunEmphasis		✓
wavelet-H_glrlm_RunEntropy		✓
wavelet-H_glrlm_RunLengthNonUniformityNormalized	✓	
wavelet-H_glrlm_RunPercentage	✓	
wavelet-H_glrlm_RunVariance		✓
wavelet-H_glrlm_ShortRunEmphasis	✓	
wavelet-H_gldm_SmallDependenceEmphasis	✓	
wavelet-H_glszm_GrayLevelNonUniformityNormalized	✓	
wavelet-H_glszm_LargeAreaEmphasis		✓
wavelet-H_glszm_SizeZoneNonUniformityNormalized	✓	
wavelet-H_glszm_SmallAreaEmphasis	✓	
wavelet-H_glszm_ZoneEntropy		✓
wavelet-H_glszm_ZonePercentage	✓	
wavelet-H_glszm_ZoneVariance		✓
wavelet-L_firstorder_10Percentile	✓	
wavelet-L_firstorder_Entropy		✓
wavelet-L_firstorder_Kurtosis	✓	✓
wavelet-L_firstorder_Minimum	✓	✓
wavelet-L_firstorder_Skewness	✓	✓
wavelet-L_glcm_Imc1	✓	✓
wavelet-L_glcm_SumEntropy		✓
wavelet-L_gldm_DependenceEntropy		✓
wavelet-L_glrlm_GrayLevelNonUniformityNormalized	✓	
wavelet-L_glrlm_LongRunLowGrayLevelEmphasis		✓
wavelet-L_glrlm_RunEntropy		✓
wavelet-L_glszm_GrayLevelNonUniformityNormalized	✓	
wavelet-L_glszm_LargeAreaLowGrayLevelEmphasis		✓
wavelet-L_glszm_ZoneEntropy		✓
wavelet-L_ngtdm_Busyness		✓
wavelet-L_ngtdm_Strength		✓
